# Higher psychological distress in patients seeking care for a knee disorder is associated with diagnostic discordance between health care providers: a secondary analysis of a diagnostic concordance study

**DOI:** 10.1186/s12891-021-04534-9

**Published:** 2021-07-30

**Authors:** Véronique Lowry, Alec Bass, Tatiana Vukobrat, Simon Décary, Patrick Bélisle, Marie-Pierre Sylvestre, François Desmeules

**Affiliations:** 1 School of Rehabilitation, Faculty of Medicine, University of Montreal, Montreal, QC Canada; 2 Orthopaedic Clinical Research Unit, Maisonneuve-Rosemont Hospital Research Center, Centre Intégré Universitaire de Santé Et de Services Sociaux de L’Est-de-L’Île-de-Montréal, 5415 Blvd L’Assomption, Pav. Rachel Tourigny, Montréal, QC H1T 2M4 Canada; 3 School of Rehabilitation, Faculty of Medicine and Health Sciences, University of Sherbrooke, Sherbrooke, QC Canada; 4 Montreal Health Innovations Coordinating Center, Montreal Hearth Institute, Montreal, QC Canada; 5 Department of Social Preventive Medicine, School of Public Health, Université de Montréal, Montreal, QC Canada

**Keywords:** Knee, Diagnosis, Concordance, Psychosocial, Depressive symptoms, Bayesian information criterion

## Abstract

**Background:**

Knee disorders are highly prevalent and may be a disabling condition. An accurate diagnosis is necessary to guide toward a rapid and efficient management of knee disorders. However, the ability to make a valid diagnosis is often complex for clinicians and evidence is mainly focused on clinician cognitive biases or errors produced during clinical reasoning. The aim of this secondary exploratory analysis is to identify patient-specific characteristics associated with diagnostic discordance between health care providers in making a diagnosis for a new knee disorder.

**Methods:**

We performed a secondary analysis of a diagnostic study comparing the diagnostic ability of a physiotherapist to medical musculoskeletal specialists. Patients’ socio-demographic, psychosocial and clinical characteristics were compared between the concordant and discordant diagnostic groups. Psychosocial symptoms were evaluated using the validated Kessler 6 (K6) questionnaire. We performed multivariable logistic regressions using the Bayesian Information Criterion to identify the most probable model including patients’ characteristics associated with diagnostic discordance. Overall probability of identified variables to explain diagnostic discordance and associated odd ratios (OR) with 95% credibility intervals (95% CrI) were calculated.

**Results:**

Overall, 279 participants were evaluated by a physiotherapist and medical musculoskeletal specialists. The mean age of the participants was 49.1 ± 15.8 years and 57.7% were female. The most common disorder was osteoarthritis (*n* = 117, 18.8% of cases were discordant). The most probable model explaining diagnostic discordance (11.13%) included having depressive symptoms, which was associated with an increased probability of diagnostic discordance (OR: 3.9; 95% CrI: 1.9 – 8.0) and having a higher number of comorbidities, which was associated with a decreased probability of diagnostic discordance (OR: 0.6; 95% CrI: 0.5 – 0.9). The depression item of the K6 questionnaire had a 99.4% chance to be included in a model explaining diagnostic discordance. Other variables taken separately had less than 50% chance to be included in a model explaining diagnostic discordance and cannot be considered significant.

**Conclusion:**

Our results suggest that depressive symptoms may increase the risk of knee diagnostic discordance. Clinicians may be more likely to make diagnostic errors and should be more cautious when evaluating patients with knee disorders suffering from psychological distress.

**Supplementary Information:**

The online version contains supplementary material available at 10.1186/s12891-021-04534-9.

## Introduction

Almost one in two adults will report knee symptoms at some time in their life [1], making knee disorders one of the most frequent reasons for consulting in primary care [2]. Knee disorders can result in important pain and have significant repercussions on a person’s ability to walk and to perform sports or vocational activities, which can lead to a decrease in health-related quality of life [3].

An early and accurate diagnosis is necessary to guide toward an efficient management of knee disorders and limit disability as well as loss of quality of life in affected individuals [4, 5]. Yet, obtaining a valid initial knee disorder diagnosis remains a common challenge [6]. The ability of primary health care providers to formulate a valid musculoskeletal diagnosis is not optimal. Evidence shows that several clinicians are often unable, based on a detailed history of the patients’ pain and physical examination tests, to accurately diagnose patients presenting with knee disorders [6, 7]. An erroneous or incomplete initial diagnosis can lead to overreliance on medical imaging tests and referrals to musculoskeletal medical specialists, thus delaying initiation of treatment [8–10]. Evidence support the fact that a comprehensive physical examination is more valid than results of medical imaging tests in a large proportion of cases [6, 11].

A valid diagnostic process requires clinical reasoning, which is defined as the cognitive process of integrating subjective and objective assessment, clinical context, as well as clinician experience, in order to make a decision regarding an optimal management strategy [12]. Many reasons may contribute to the failure of this process [13]. Research has mainly focused on clinician cognitive biases or errors produced during clinical reasoning [14–18]. We also know that some factors related to patients such as a lack of cooperation or unusual disease presentation may influence the ability of health care providers to make a valid diagnosis [19]. Other factors such as patients’ comorbidities, psychological distress and fear-avoidance beliefs are associated with worse pain, function and health-related quality of life and could influence the diagnostic process as well [20, 21]. However, to our knowledge, no study has looked at patient-specific characteristics, such as socio-demographic, psychosocial and clinical characteristics that could potentially affect the diagnostic process for patients presenting with knee disorders [14–16, 22].

In a previous study undertaken by our team, diagnostic concordance between a physiotherapist and medical musculoskeletal specialists was found to be high for patients with various knee disorders [23–27]. The present study is a secondary exploratory analysis of this cohort of patients and aims to understand factors affecting knee diagnostic concordance or its contrary discordance. In our exploratory analysis, we hypothesized that diagnostic discordance between two health care providers is a reflect of the difficulty to make a valid diagnosis and therefore a proxy of increased risks of diagnostic error. The objective of this study was to identify potential patients’ specific characteristics associated with diagnostic discordance between medical musculoskeletal specialists and a physiotherapist in making a diagnosis for a knee disorder.

## Methods

### Study setting

We conducted a secondary analysis of a multicenter prospective diagnostic study taking place in two outpatient orthopaedic clinics and two primary care family medicine clinics in Montreal and Quebec City, Canada. The original study aimed to assess the diagnostic validity of various clusters combining history elements and physical examination tests to diagnose common knee disorders [23–27].

Patients were recruited at their initial consultation with a medical musculoskeletal specialist (sport medicine physicians or orthopaedic surgeons) when seeking care for a knee disorder. Also, student and personnel from a University community (Laval University, Quebec City, Canada) seeking care for a current knee complaint were invited by email to participate in the original study. The study was explained to all participants and a written informed consent was obtained prior to the consultation. Participants were advised that they could withdraw at any time without prejudice and without affecting the quality of care they would receive. The study protocol was approved by the ethic committees of the Centre intégré universitaire de santé et services sociaux (CIUSSS) de l’Est-de-l’Île-de-Montréal and the CIUSSS de la Capitale-Nationale. All procedures were followed in accordance with relevant ethical guidelines.

### Participants

Participants were included in the study if they were: 1–18 years of age or older, 2- consulting for a knee complaint, 3- able to speak or understand French, 4- a resident of the province of Quebec and covered by the provincial health insurance (Régie de l’assurance maladie du Québec) and 5- legally able to consent. Participants who had already consulted one of the medical musculoskeletal specialists for a knee disorder were excluded. Also, patients that had undergone lower limb surgery in the 6 months preceding recruitment or with a previous knee arthroplasty were excluded. Finally, participants presenting with more than two others known concomitants pathologies of the lower limb and/or for a knee disorder associated with a systemic inflammatory disorder were not eligible for this study.

### Patients’ socio-demographic, psychosocial and clinical characteristics

Participants answered a standardized questionnaire which included information on gender, age and anthropometric data to allow calculation of body mass index (BMI). We also recorded their education level, employment status and number of comorbidities (osteoarthritis (OA) in other joints, hearth disease, high blood pressure, diabetes). The duration of knee symptoms, affected side, knee pain location (anterior, posterior, medial, lateral or diffuse), presence of bilateral knee pain, onset mechanism (traumatic or progressive), timing of symptoms onset, apparition of joint swelling if the patient reported a traumatic onset mechanism and current use of a walking aid were also recorded. Participants had to indicate if they were seeking care for their knee disorder for the first time. Participants also completed the validated French version of the Knee Injury and Osteoarthritis Outcome Score (KOOS), a self-reported 42-item questionnaire that assesses pain, symptoms, function in daily living, function in sport and recreation and knee-related quality of life [28–30]. The KOOS score includes five subscale: symptoms, pain, activity of daily living function, sports and recreation function and quality of life [31]. Each subscale is calculated on a 0–100 scale with higher score indicating lower disability [31]. Psychological distress was assessed using the validated French version of the Kessler-6 (K6) screening scale for serious mental disorders [32–34]. The K6 includes six items rated between 0 (None of the time) and 4 (All of the time). The questions aim at evaluating if the participant is feeling nervous, hopeless, restless, depressed, worthless or feels that everything is an effort over the last 30 days. The K6 is validated to screen for depressive and anxious symptoms [35]. A K6 score equal or above 5 indicates moderate mental distress and a K6 total score indicating severe psychological distress is equal or above a score of 13 [36].

### Data collection, initial diagnosis and definition of concordance

After completion of the questionnaires, the participants were independently evaluated by a physiotherapist and one of the five musculoskeletal medical specialists (three orthopaedic surgeons and two sport medicine physicians). One physiotherapist performed all examination. The physiotherapist had a professional master’s degree and 1 year of clinical experience and the musculoskeletal medical specialists each had more than 20 years of clinical experience. The two evaluations were separated by a maximum of 15 min. First, the physiotherapist performed the physical examination of the participants following a standardized examination. The standardized examination included elements such as observation, functional tests, special knee tests and palpatory exam (described in Additional file 1). Then, the medical musculoskeletal specialist proceeded to his independent history taking and physical examination. Medical imaging results for all participants were also collected, but only the medical musculoskeletal specialist had access to this information at the time of the clinical examination. Both the physiotherapist and the medical musculoskeletal specialist were blinded to each other results.

After independently seeing the patient, each evaluator completed a separate standardized form where they indicated their primary and, when applicable their secondary diagnosis. The physiotherapist made a primary and secondary diagnosis (if necessary) based only on his clinical assessment. The medical musculoskeletal specialist’s primary and secondary diagnosis (if necessary) was based on his clinical assessment and his interpretation of the results of available diagnostic imaging. The evaluators did not have access or used the KOOS and K6 questionnaires during their examination. All participants were required to have an X-ray of their knee, which was clinically required for a consultation at the orthopaedic outpatient clinic. Magnetic resonance imaging (MRI) was required for all suspected ligament tears, meniscal tears, or to exclude any other knee diagnosis. Each diagnosis was classified into one of the following categories: meniscal tears, patellofemoral pain, anterior cruciate ligament (ACL) tear, knee OA or other knee pathology. The diagnoses made by both evaluators were classified as concordant or discordant. To be classified as a concordant case, the evaluators had to indicate the primary and the secondary diagnosis (when applicable), they had to be identical and to be in the same order (primary and secondary).

### Statistical analysis

Descriptive statistics were used to present participants’ socio-demographic, psychosocial and clinical characteristics according to diagnostic concordance or discordance in the cohort of patients seeking care for a knee disorder. All variables included in the analyses are listed in Additional file 2.

To evaluate if participants’ characteristics between the concordant and discordant diagnostic groups according to the definition of concordance are significatively different, student t-tests were used for continuous variables and chi-square tests were used for categorical variables. We compared the distribution of both the K6 total score and its six items (nervousness, hopelessness, restlessness, depression, feeling that everything is an effort, worthlessness) between concordant and discordant diagnostic groups. We recoded the K6 items as binary indicators of psychosocial symptoms for sensitivity analyses. Response choice corresponding to a little of the time, some of the time, most of the time or all of the time were coded as having symptoms. Participants’ characteristics that were statistically different between concordant and discordant groups were also analyzed using simple logistic regression and odd ratios (OR) with 95% confidence intervals (95% CI) were calculated for the association between the socio-demographic, psychosocial and clinical characteristics of the participants and diagnostic discordance. These statistical analyses were performed using IBM SPSS Statistics Version 25.

Multivariable logistic regression was then used to estimate association between selected independent variables and discordant cases. The Bayesian Information Criterion (BIC) was used for model selection to identify sets of variables that best explained diagnostic discordance [37, 38]. The posterior probability that each model including independent variables explained diagnostic discordance (model probability) was estimated and reported. The associated probability that the included independent variables taken separately were present in a model explaining diagnostic discordance (probne0) was also estimated. Variables were included in the model with an initial probability to be present in the model (probne0) of 50%. Therefore, if the data went in favor of a given variable, its posterior probability (probne0) was above 50% [38]. However, any variable with posterior probability lower than 80% is often reported in the literature as not being important [39]. The five most probable models according to the BIC with their respective probability to explain diagnostic discordance (model probability) were then presented with associated odds ratio (OR) and 95% credibility interval (95% CrI). We also presented the total probability that the variables taken separately explained diagnostic discordance (probne0). These statistical analyses were performed using R Version 3.4.2.

## Results

### Participants

**Fig. 1:**
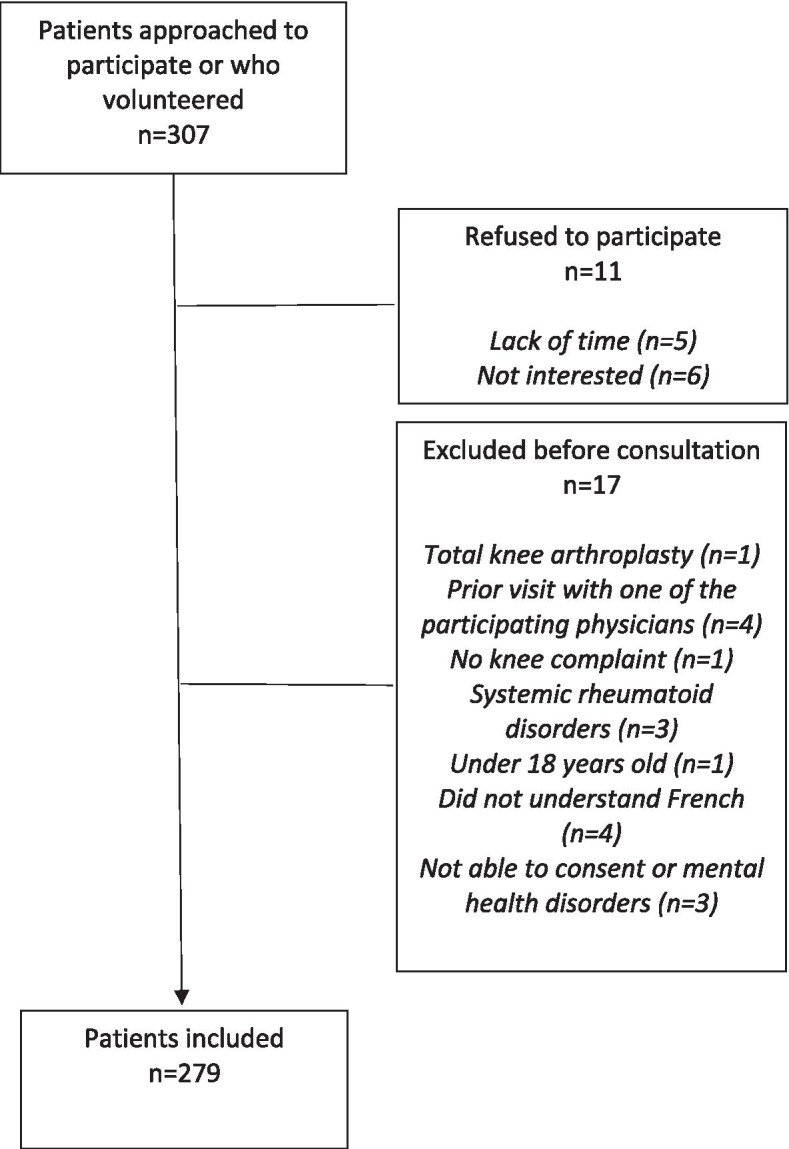
Flow chart of patients’ recruitment

Two hundred seventy-nine participants consulting for knee disorders were recruited (Fig. 1). The mean age of the participants was 49.1 (SD: 15.8) years and the majority were woman (57.7%). The most common diagnosis was knee OA (41.9%) and 68.8% of the participants had a progressive symptom onset. Overall concordance was observed for 201 participants (72%). Table 1 presents the socio-demographic and clinical characteristics according to a concordant or discordant diagnosis and Table 2 presents the psychosocial characteristics of the participants as measured with the K6 (*n* = 259). Thirty-three (32.8%) percent of the participants had moderate mental distress and 5.0% of the participants had severe mental distress.

**Table 1:** Socio-demographic and clinical characteristics of participants consulting for a knee complaint according to diagnostic concordance between health providers (*n* = 279)

	**Discordant diagnosis (*n* = 78)**		**Concordant diagnosis (*n* = 201)**		**Between-group differences (p)**	**OR (95% CI)**	***P***
	**n (%)**	**Mean (SD)**	**n (%)**	**Mean (SD)**			
**Age**		45.7 (13.6)		50.4 (16.5)	0.03*	0.98 (0.96–0.998)	0.03*
**Gender**							
**Woman**	46 (59.0)		115 (57.2)		0.79		
**BMI (kg/m2)**		29.5 (6.5)		29.3 (6.6)	0.81		
**Employment status**							
Employed	53 (67.9)		121 (60.2)		0.008*	1.4 (0.8–2.4)	0.2
Sick leave	12 (15.4)		14 (7.0)			2.4 (1.1–5.5)	0.03*
Retired	5 (6.4)		42 (20.9)			0.26 (0.10–0.68)	0.006*
Unemployed	8 (10.3)		24 (11.9)			0.84 (0.36–2.0)	0.7
**Work physical demand**							
1/ Sitting	20 (25.6)		63 (31.3)		0.02*	1.2 (1.0–1.5)	0.08
2/ Light	12 (15.4)		20 (10.0)				
3/ Moderate	0		0				
4/ High	31 (39.7)		51 (25.4)				
**Duration of symptoms**							
≤ 1 month	7 (9.0)		10 (5.0)		0.21		
> 1 month	71 (91.0)		191 (95.0)				
**Onset Mechanism**							
1/ Traumatic	35 (44.9)		52 (25.9)		0.002*	0.43 (0.25–0.74)	0.002*
2/ Progressive	43 (55.1)		149 (74.1)				
**Pain Location**							
Diffuse	33 (42.3)		81 (40.3)		0.76		
Located	45 (57.7)		120 (59.7)				
**Use of WA**							
**Yes**	8 (10.3)		21 (10.4)		0.96		
**First consultation**							
**Yes**	22 (28.2)		37 (18.4)		0.07		
**Personal income (*n* = 177)**							
≤ 30 000$	23 (47.9)		69 (53.5)		0.51		
> 30 000$	25 (52.1)		60 (46.5)				
**Timing of symptoms onset if traumatic onset**							
Pain appeared immediatly	30 (85.7)		47 (95.9)		0.1		
Pain appeared with a delay	5 (14.3)		2 (4.1)				
**Timing of apparition of joint swelling if present**							
< 2 h after trauma	13 (43.3)		18 (45.0)		0.9		
> 2 h after trauma	17 (56.7)		22 (55.0)				
**Bilateral symptoms:**							
1/ No	70 (89.7)		156 (77.6)		0.02*	0.40 (0.18–0.89)	0.02*
2/ Yes	8 (10.3)		45 (22.4)				
**Number of comorbidities**		0.7 (0.9)		1.0 (1.0)	0.01*	0.69 (0.51–0.93)	0.02*
**MRI results available at the time of consultation**	43 (55.1)		72 (35.8)		0.003*	2.2 (1.3–3.7)	0.004*
**Diagnosis**							
Knee Osteoarthritis	22 (28.2)		95 (47.3)		< 0.001*	0.44 (0.25–0.77)	0.004*
Patellofemoral syndrome	15 (19.2)		44 (21.9)			0.85 (0.44–1.64)	0.6
Meniscal injury	16 (20.5)		38 (18.9)			1.1 (0.6–2.1)	0.8
ACL injury	10 (12.8)		17 (8.5)			1.6 (0.7–3.6)	0.3
Other knee injury^a^	15 (19.2)		7 (3.5)			6.6 (2.6–16.9)	< 0.001*
**Number of diagnoses**							
1/ One diagnosis	43 (55.1)		156 (77.6)		< 0.001*	2.8 (1.6–4.9)	< 0.001*
2/ Two or more diagnoses	35 (44.9)		45 (22.4)				
**KOOS Symptoms (/100)**^**b**^		72.2 (17.2)		69.2 (19.8)	0.26		
**KOOS Pain (/100)**^**b**^		58.6 (19.9)		58.5 (20.0)	0.97		
**KOOS Activities of daily living function (/100)**^**b**^		65.6 (22.1)		66.0 (22.0)	0.89		
**KOOS Sports and recreation function (/100)**^**b**^		25.3 (22.6)		30.0 (26.1)	0.17		
**KOOS Quality of life (/100)**^**b**^		38.1 (20.2)		38.6 (19.7)	0.86		

*X* Mean, *SD* Standard deviation, *OR* Odd ratio (associated to diagnostic discordance), *95% CI* Confidence Interval, *p* Level of significance, *BMI* Body mass index, *WA* Walking aid, *First consultation* First consultation for knee pain or disorder, *KOOS* Knee Injury and Osteoarthritis Outcome Score

^*^p < 0,05

^a^Other knee injury include all diagnosis that could not be classified in the following categories: Knee Osteoarthritis, Anterior Cruciate Ligament injury, Meniscal injury, Patellofemoral Syndrome

^b^A higher score on the KOOS scale indicates lower disability

**Table 2:** Psychosocial characteristics of participants consulting for a knee complaint according to diagnostic concordance between health providers (*n* = 259^a^)

	**Overall (*n* = 259)**	**Overall (*n* = 259)**	**Overall (*n* = 259)**	**Discordant diagnosis (*n* = 70)**	**Concordant diagnosis (*n* = 189)**	**Student t-test**^**b**^** (*p*-value)**	**Chi-square test**^**c**^** (*p*-value)**	**Recoded questions in categories**^**d**^	**Discordant diagnosis (*n* = 70)**	**Concordant diagnosis (*n* = 189)**	**Chi-sqare test (*p*-value)**	**OR (95%CrI)**	***P*-value**
	**X (SD)**	**Median (range)**	**Mode**	**X (SD)**	**X (SD)**				**n (%)**	**n (%)**			
K6 total score (/24)				4.8 (5.4)	3.2 (4.2)	0.01†						1.1 (1.01–1.1)	0.02†
Nervous (/4)	1.3 (1.3)	1 (0–4)	0	1.5 (1.3)	1.2 (1.3)	0.17	0.09	Nervous (Yes)	51 (72.9)	109 (57.7)	0.03†	2.0 (1.1–3.6)	0.03†
Hopeless (/4)	0.5 (1.0)	0 (0–4)	0	0.7 (1.1)	0.4 (0.9)	0.02†	0.00†	Hopeless (Yes)	30 (42.9)	39 (20.6)	0.00†	2.9 (1.6–5.2)	0.000†
Restless (/4)	0.8 (1.1)	0 (0–4)	0	0.9 (1.2)	0.7 (1.1)	0.12	0.01†	Restless (Yes)	36 (51.4)	61 (32.3)	0.005†	2.2 (1.3–3.9)	0.005†
Depressed (/4)	0.3 (0.8)	0 (0–4)	0	0.4 (0.9)	0.2 (0.7)	0.04†	0.001†	Depressed (Yes)	20 (28.6)	21 (11.1)	0.001†	3.2 (1.6–6.4)	0.001†
Everything is an effort (/4)	0.6 (1.1)	0 (0–4)	0	0.8 (1.2)	0.5 (1.0)	0.02†	0.07	Everything is an effort (Yes)	30 (42.9)	48 (25.4)	0.007†	2.2 (1.2–3.9)	0.007†
Worthless (/4)	0.2 (0.7)	0 (0–4)	0	0.4 (0.9)	0.2 (0.6)	0.02†	0.03†	Worthless (Yes)	17 (24.3)	18 (9.5)	0.002†	3.0 (1.4–6.3)	0.003†

*OR* Odd ratio, *CrI* Credibility interval

^†^*p* < 0.05

^a^*n* = 259; 20 participants refused to answer the K6 questionnaire for personal reasons

^b^For continuous variables

^c^For categorical variables

^d^The K6 questions were recoded in two categories as no symptom or presence of the symptom (A little of the time, some of the time, most of the time all of the time)

### Univariate analyses

There were statistically significant differences between the concordant and discordant groups for nine characteristics of the participants: age (*p* = 0.03), employment status (*p* = 0.008), work physical demand (*p* = 0.02), symptom onset mechanism (*p* = 0.002), bilateral or unilateral involvement (*p* = 0.02), number of comorbidities (treated as a continuous variable) (*p* = 0.01), having a MRI result at the time of consultation (*p* = 0.003), according to types of diagnosis (*p* < 0.001) and number of diagnoses (*p* < 0.001). There was also a statistically significant difference between groups for the K6 total score (*p* = 0.01). When recoded as a dichotomous variable (having psychosocial symptoms or no symptoms) there were statistically significant differences between groups for all six items of the K6 (*p* < 0.05) (Table 2).

Based on univariate logistic regression analyses, five characteristics were associated with an increased probability of diagnostic discordance: being on sick leave (OR: 2.4; 95% CI: 1.1—5.5), having MRI results available at the time of consultation (OR: 2.2; 95% CI: 1.3—3.7), having a diagnosis classified as an “other knee disorder” after evaluation (OR: 6.6; 95% CI: 2.6—16.9) and having more than one final knee diagnosis (OR: 2.8; 95% CI: 1.6—4.9) (Table 1). Having a higher score on the K6 questionnaire was also associated with a higher probability of diagnostic discordance (OR: 1.1; 95% CI: 1.01—1.1). All recoded questions of the K6 (in categories) were also associated with an increased probability of diagnostic discordance with OR ranging from 2.0 to 3.2 (Table 2).

Six characteristics of the participants were associated with an increased probability of diagnostic concordance (Table 1). Older age of the participant (OR: 0.98; 95% CI: 0.96—0.998), being retired (OR: 0.26; 95% CI: 0.10—0.68), having a progressive onset of symptoms (OR: 0.43; 95% CI: 0.25—0.74), having pain in both knees (OR: 0.40; 95% CI: 0.18—0.89;), having a higher number of comorbidities (OR: 0.69; 95% CI: 0.51—0.93) and having a knee OA diagnosis (OR: 0.44; 95% CI: 0.25—0.77) were associated with an increased probability of diagnostic concordance.

### Multivariate analysis

Because of missing values for some independent variables, 259 participants were included in the multivariate logistic regression analysis using the BIC approach. Personal income was not included in the analysis because of a high number of missing values (*n* = 102). Results are presented in Table 3. The most probable model (11.13%) includes the recoded K6 depression question as a dichotomous variable and the number of comorbidities. In this model, having depressive symptoms in the last 30 days as measured with the depression question of the K6 (feeling so depressed that nothing could cheer you up at some time) is associated with an increased probability of diagnostic discordance (OR: 3.9; 95% CrI: 1.9 – 8.0) and having a higher number of comorbidities is associated with a decreased probability of diagnostic discordance (OR: 0.6; 95% CrI: 0.5 – 0.9).

**Table 3:** Most probable models explaining discordance with Bayesian Information Criterion (*n* = 259)

		Variables	**k6 Depression**	**MRI**	**Comorbidities**	**Progressive symptoms**	**KOOS_Symptoms**	**First consultation**
		probne0	99.4%	44.5%	42.6%	33.4%	30.5%	27.4%
**Rank**	**Model probability**	**Model description**	**OR (95% CrI)**	**OR (95% CrI)**	**OR (95% CrI)**	**OR (95% CrI)**	**OR (95% CrI)**	**OR (95% CrI)**
1	11.13%	Comorbidites, k6 Depression	3.9 (1.9–8.0)		0.6 (0.5–0.9)			
2	7.60%	MRI, first consultation, k6 Depression	3.9 (1.9–8.2)	2.4 (1.3–4.3)				2.3 (1.2–4.5)
3	6.88%	MRI, k6 Depression	3.8 (1.8–7.8)	2.2 (1.2–4.0)				
4	6.86%	Progressive symptoms, k6 Depression	3.2 (1.6–6.5)			0.5 (0.3–0.8)		
5	6.53%	MRI, Comorbidites, k6 Depression	4.3 (2.1–9.1)	1.9 (1.0–3.5)	0.7 (0.5–1.0)			

*Model probability* Probability that the model explain diagnostic discordance, *probne0* Probability that the variable is included a model explaining diagnostic discordance, *OR* Odd ratio of the variable explaining diagnostic discordance in the model, *95% CrI* 95% Credibility Interval, *k6 Depression* Having self-reported depressive symptoms on the depression item of the k6 (Yes or No), *MRI result* Having an MRI result and an X-Ray at the time of the consultation compared to only X-ray, *KOOS_Symptoms* Knee Injury and Osteoarthritis Outcome Score Symptom Scale, *First consultation* First consultation for knee pain or disorder

The second most probable model (7.60%) also includes the recoded K6 depression item as a dichotomous variable, having an MRI result at the time of consultation and if the patient consults for the first time for the current knee complaint. In this model, having depressive symptoms (OR: 3.9; 95% CrI: 1.9–8.2), having MRI results at the time of consultation (OR: 2.4; 95% CrI: 1.3 – 4.3) and consulting for the first time for a knee complaint (OR: 2.3; 95% CrI: 1.2 – 4.5) are associated with an increased probability of diagnostic discordance. Other less probable models as a result from the BIC approach are also presented in Table 3.

The probability that the recoded depression item of the K6 is included in a BIC model explaining diagnostic discordance (probne0) is 99.4%. In every possible model, having depressive symptoms is associated with an increased probability of diagnostic discordance with ORs ranging between 3.2; 95% CrI 1.6 – 6.5 and 4.3; 95% CrI 2.1 – 9.1 in the five most probable models. Other variables taken separately had less than 50% chance to be included in a model explaining diagnostic discordance and cannot be considered significant (Table 3).

## Discussion

The aim of this exploratory analysis was to identify potential patients’ specific characteristics associated with diagnostic discordance between medical musculoskeletal specialists and a physiotherapist for patients presenting with a knee complaint. We have hypothesized that diagnostic discordance is a consequence of the difficulty to evaluate and make a valid and reliable diagnosis for a patient [40], and that it can be considered a proxy of diagnostic errors. Diagnostic errors can have important detrimental effects on the patient’s life by affecting management and contributing to inefficient use of healthcare resources [8, 10, 41, 42]. Since results from univariate logistic regression must be interpreted cautiously because of the possible confounding factors [43], we also used multiple logistic regression with the BIC approach to select and evaluate the best models that includes patients’ characteristics that could explain diagnostic discordance between evaluators.

The K6 recoded depression question as a dichotomous variable had a probability of 99.4% to be included in a model explaining diagnostic discordance. In the five most probable models based on multivariate logistic regression, having depressive symptoms was associated with 3.2 to 4.3 times more chance of diagnostic discordance. It is important to note that total K6 score and all separate questions of the K6 (treated as ordinal variables either in the original scale or as dichotomous variables (having the symptom or not) were also associated with a higher probability of diagnostic discordance in our univariate analyses.

Our results suggest that a patient’s psychological distress is associated with diagnostic discordance between musculoskeletal experts or evaluators even in the presence of overall high diagnostic agreement between evaluators in this cohort of patients [23–27]. Recent reviews have demonstrated that depression and pain symptoms are highly prevalent conditions encountered by primary care physicians and specialists, with 30 to 60% co-occurrence rate [44, 45]. In our study, 32.8% percent of the participants had moderate mental distress and 5.0% of participants had severe mental distress as measured with the K6. Recent studies have found that comorbid depressive symptoms significantly influences the severity of knee pain [21, 46–49], which is also a predictor of depression severity [45]. A recent systematic review and meta-analysis has found a significant correlation between pain severity and levels of anxiety and depression in osteoarthritis patients [20]. However, our analysis does not allow to prove a causal relationship between knee pain and depressive symptoms. Depressive symptoms have been associated with an increase in the severity of reported knee pain, likely because of increased cytokine levels and neurotransmitters’ changes related to depression, which are known to influence the threshold of physical pain perception [50]. Also, pain descending inhibitory pathways may be affected with depression, thus influencing the modulation of pain [51]. Therefore, when patients have depressive symptoms, pain may be less explained by nociception or a specific biological lesion [52]. Moreover, in patients with psychological distress, pain behavior can be modified, which can influence symptomatology [52, 53]. These mechanisms could influence the results of physical examinations which often are a combination of pain provocative tests that aim at identifying a pathoanatomical lesion. The pain provocative tests may therefore be less accurate in that situation, affect the assessment’s outcomes and increase the risk of discordance or of an inaccurate diagnosis. Depressive disorders are also associated with cognitive impairments such as memory and attention loss, which may have affected the history taking and thus may induce errors in the diagnostic process [54].

Despite its potential impact on patient’s severity of symptoms, the diagnostic process and overall management, psychological distress assessment is not routinely performed by clinicians in musculoskeletal disorders care [55]. Clinicians may need to be more cautious when evaluating and treating patients with musculoskeletal disorders presenting with psychological distress. Bio-psycho-social models of care such as those that have been developed for the management of low back pain [52] and routine identification of psychosocial symptoms [56] with questionnaires such as the K6 may need to be more systematically considered and promoted for the evaluation and treatment of knee or other musculoskeletal disorders.

Other variables that were included in other models explaining diagnostic discordance may also be of interest, although the BIC threshold of 50% was often not achieved and these variables cannot be considered significant in explaining diagnostic discordance. Having an MRI result available at the time of consultation, having more disability on the KOOS Symptoms Scale, and consulting for the first time for a knee complaint were all associated with a higher probability of diagnostic discordance.

Having a higher number of comorbidities and having a progressive onset of symptoms was associated with a lower probability of diagnostic discordance. Some of those variables were significantly associated with the probability of diagnostic discordance in the univariate analysis results as well. Having a progressive onset of symptoms and having a higher number of comorbidities were associated with the decreased probability of diagnostic discordance in univariate analysis. These findings could be explained by the fact that these clinical characteristics are common in patients with knee OA [57–60]. Having a diagnostic of knee OA was associated with a decreased probability of diagnostic discordance as well in our study. Knee OA may be easier to diagnose since older age of the patient is a good indicator of OA [26]. Having MRI results, which was associated with a higher probability of diagnostic discordance in both analyses, could be related to more complex traumatic injuries and reveal concomitant lesions, although this was not shown in our results. The diagnostic imaging results were only available to the musculoskeletal medical specialists and MRI of the knee often reveal asymptomatic lesions which may not be highlighted with physical examination [61, 62]. After looking at the MRI results, the medical musculoskeletal specialist may have indicated a secondary diagnosis based on these results, whereas the physiotherapist was not influenced by those results and may not have associated the lesion revealed by the imaging test with the patient’s symptoms.

### Strengths and limitations

To our knowledge, this study is the first to explore and evaluate factors explaining diagnostic discordance for musculoskeletal disorders between health care providers with specialized training in musculoskeletal disorders care. The analysis was performed using a database of a prospective diagnostic study including an extensive set of socio-demographic, psychosocial and clinical variables and rigorous statistical analyses were performed. However, the exploratory nature of the study and the use of an existent database have limitations. The diagnosis was compared between different health care providers with the medical musculoskeletal specialists’ final diagnosis including the diagnostic imaging results as a reference standard and this may be seen as a limitation. However, this reference standard is often used when no gold standard exists and represents a real diagnostic process in the clinical setting [63]. Also, the medical musculoskeletal specialists used the results of diagnostic imaging to make their diagnoses, but the physiotherapist did not have access to these results when performing his assessment. This situation may have influenced diagnostic concordance, but this variable was only found significant in one of the proposed models. The number of providers performing the evaluations was low which may limit the generalizability of the results. However, the study took place in four different settings and different type of medical musculoskeletal specialists evaluated the patients.

## Conclusion

The results of this secondary exploratory analysis suggest that the most important patient-specific characteristics associated with a higher risk of diagnostic discordance between a physiotherapist and medical musculoskeletal specialists is the presence of psychological distress. It is likely that patients’ psychosocial and clinical characteristics may alter symptoms and clinical presentation of knee disorders, which could diminish the ability to make a valid diagnosis. Clinicians may be more likely to make diagnostic errors and should be more cautious when evaluating patients with knee disorders suffering from psychological distress. More research is needed to fully understand and validate these results, but clinicians may need to consider pain as a biopsychosocial experience when evaluating and treating patients with musculoskeletal disorders [52].

## Supplementary Information

**Additional file 1.** Standardized Examination Guide.

**Additional file 2.** List of analyzed variables.

## Data Availability

The datasets used and/or analyzed during the current study are available from the corresponding author on reasonable request.

## Abbreviations

K6: Kessler 6
OR: Odd ratios
95% CrI: 95% Credibility intervals
CIUSSS: Centre intégré universitaire de santé et services sociaux
BMI: Body mass index
OA: Osteoarthritis
KOOS: Knee Injury and Osteoarthritis Outcome Score
MRI: Magnetic resonance imaging
ACL: Anterior cruciate ligament
95% CI: 95% Confidence intervals
BIC: Bayesian Information Criterion
probne0: The associated probability that the included independent variables taken separately was present in a model explaining diagnostic discordance
