# Supplementary material for: Higher psychological distress in patients seeking care for a knee disorder is associated with diagnostic discordance between health care providers: a secondary analysis of a diagnostic concordance study
Source: BMC Musculoskelet Disord. 2021 Jul 30;22:650. doi: 10.1186/s12891-021-04534-9 (PMC8325325; doi:10.1186/s12891-021-04534-9)
Supplement: Supplementary file 1 — Additional file 1. Standardized Examination Guide. [file 12891_2021_4534_MOESM1_ESM.docx]

| **Additional file 1: Standardized Examination Guide** | |  |
| --- | --- | --- |
| TESTS | DESCRIPTION | INTERPRETATION |
| PALPATORY EXAM |  |  |
| Palpatory examination of the different structures of the knee. | Perform a palpatory examination of the following structures: quadricipital tendon, patellar tendon, medial and lateral joint spaces, patellar apex, medial and lateral patellar facets, fibula head, crow's feet, popliteal fossa, sembranous, semi-tendinous tendons and femoral biceps. Compare between the affected and healthy knee to verify that the excessive pressure is not causing a false positive. For palpation of the joint space, ensure that the knee is at 90 degrees of flexion to standardize this test. | **Positive:** the participant said that he experienced pain / discomfort when pressing on the structure. The pain / discomfort should reproduce that for which the participant consulted (to be differentiated from pain due to pressure, but which the patient does not recognize). A spasm is also interpreted as a positive test. **Negative:** the participant said that he did not feel any pain when he pressed on the structure. Evidence of discomfort that is not representative of their Sy is considered to be negative. |
| Presence of heat (OA and inflammation) | During the palpatory examination, determine if there is heat in the region of the joint. Compare to the healthy knee. Compare with the temperature at the thigh. | **Positive:** during palpation, you generally feel a heat in the knee and this is different from the healthy knee. **Negative:** you don't feel any difference in temperature between the two knees. |
| Presence of cracking. | Place your hand on the knee to cover the patella and as much surface as possible. Hold the joint space between the 1st and 5th metacarpus. Compare both knees to avoid a false positive. You can determine the presence of cracklings in four situations: during the mobilization of the patella (sign of the plane), during active flexion / extension or during passive flexion / extension, when the patient stands up (loading). | **Positive:** you feel or hear vibrations characteristic of crackings. **Negative:** you do not feel any cracking. |
| OBSERVATION |  |  |
| Limitation of the passive range of motion in flexion and extension. | Ask the participant to perform a full flexion followed by a full knee extension with the healthy lower limb followed by the affected knee. The participant must return in extension between the two movements to avoid pressure on the back. Then passively perform a full flexion followed by full knee extension with the healthy lower limb followed by the affected knee. | **Positive**: you observe that the participant is not able to make an equivalent flexion / extension between the healthy and affected lower limb. You observe that the passive amplitude in flexion / extension is not equivalent between the healthy and affected lower limb.  **Negative:** you observe a similar amplitude between the two lower limbs for active and passive flexion / extension. |
| Painful isometric resistant extension at 90 ° and 30 ° of flexion | The participant is in a seated position. Place the affected lower limb in the 90 ° flexion position. Place your hand at the participant's ankle and ask them to try to make a knee extension by forcing against you. Resist so that the limb does not move. | **Positive:** the participant does feel pain or discomfort in the knee when the contraction was resisted. Must represent the usual Sy.  **Negative:** the participant does not feel any pain when the contraction is resisted or the discomfort is not representative of the usual Sy. |
| Visual evaluation of the morphology of the lower limbs | The participant is in standing position with his feet stick together. Observe the morphology of its lower limbs. Select the morphological variations observed. | **Choose from the following variations:** knee valgus, knee varus, knee recurvatum, normal, uncertain |
| Visual assessment of patellar morphology | The participant is standing in a comfortable position. Observe the morphology of its patellas. Select the morphological variations observed. | **Choose from the following variations:** outward facing, inward facing, patella alta, normal, uncertain |
| SPECIFIC TESTS FOR KNEE INFLAMMATION | |  |
| Stroke sign test (4-8 ml extra fluid) | The participant is in supine position. With one hand placed medial to the knee, make 2-3 "brush" movements upwards, then make a brush movement downwards with the other hand placed outside the knee. Wait two seconds to ensure the movement of fluids. You can perform the maneuver up to 2-3 consecutive times before interpreting the test. | **Positive:** you observe a slight swelling that appears under the medial aspect of the patella. Swelling around the patellar tendon (medial or lateral) is also considered a positive test. |
| Patellar tap test (40-50 ml extra fluids) | The participant is in supine position. You place one hand above the knee and the other hand below the knee. You bring both your hands towards the knee. Using your thumb, push the patella toward the trochlea and watch the patella return to its original position. | **Positive:** you observe that the patella seems to "float" to its original position. |
| SPECIFIC TESTS FOR LIGAMENTS INJURIES | |  |
| Lachman | The participant is in supine position. Bend the knee between 15 and 30 degrees of flexion. Stabilize the distal femur with one hand, while your other hand grips the proximal tibia from the back. Apply anterior force to pull the shin forward. Force must be produced quickly and of short duration. | **Positive:** the tibia produces a greater anterior displacement on the affected side than on the healthy side. |
| Anterior drawer test | The participant is in supine position. Bend the knee at 90 degrees, sit on the participant's foot and grasp with both hands the proximal tibia with your thumbs on the anterior tibial trays and the index fingers palpating the hamstring tendons in the posterior. Apply anterior force to pull the shin forward. Force must be produced slowly. | **Positive:** the tibia produces a greater anterior displacement on the affected side than on the healthy side. |
| Pivot shift test | The participant is in supine position. Place the knee in full extension and then ask the participant to completely relax his muscles (the test is difficult to interpret if the participant produces co-contraction). Firmly grad proximally and distally the tibia and perform an internal rotation of the lower limb. Holding this internal rotation position, slowly initiate passive knee flexion. | **Positive:** you observe an external subluxation of the tibial plateau from the start of the flexion or palpate a dull sound / vibration or a "click". You can perform the maneuver a few times depending on the participant's tolerance before deciding on the test result. |
| Posterior drawer test | The participant is in supine position. Bend the knee at 90 degrees, sit on the participant's foot and grasp with both hands the proximal tibia with your thumbs on the anterior tibial plateau and the index fingers palpating the hamstring tendons in the posterior. Apply posterior force to push the tibia back. Force must be produced slowly. | **Positive:** the tibia produces greater posterior displacement on the affected side than on the healthy side. |
| Internal collateral ligament test at 0 and 30 degrees | The participant is in supine position. Bend the knee to 0 degrees (full extension) and firmly grasp the tibia between your arm and the side of your body, you are placed outside of the participant's lower limb. Place the other hand distal to the femur, on the outside of the knee. The external knee hand applies a slight medial force while the other arm performs a slight external rotation of the tibia simultaneously. Repeat the test at 30 degrees. | **Positive:** an excessive medial opening compared to the healthy side and / or the participant complains of pain / discomfort representative of Sy during the test. |
| External collateral ligament test at 0 and 30 degrees | The participant is in supine position. Bend the knee to 0 degrees (full extension) and firmly grasp the tibia between your arm and the side of your body, you are placed internally of the participant's lower limb. Place the other hand distal to the femur, on the inside of the knee. The internal knee hand applies a slight lateral force while the other arm performs a slight external rotation of the tibia simultaneously. Repeat the test at 30 degrees. | **Positive:** an excessive lateral opening compared to the healthy side and / or the participant complains of pain / discomfort representative of Sy during the test. |
| Specific tests for meniscal injuries | The participant is in supine position. Take the lower limb at the heel while the other hand grasps the knee between the thumb and forefinger to palpate the medial and lateral joint space. Passively perform a complete knee flexion. To test the medial meniscus, use the lower hand to rotate the tibia externally and then slowly extend the knee. To test the lateral meniscus, perform an internal rotation of the tibia and then slowly extend the knee. You can perform a "scooping" movement in full flexion in order to try to bring out the "click" and then continue with the extension. You must be able to flex at least almost completely to interpret this test. | **Positive:** if you hear or feel a matt or "click" sound / vibration. As for the Thessaly, we add that a pain representative of the patient's Sy during the maneuver is considered to be positive (pain in torsion). On the other hand, the pain from overpressure on scooping in full flexion does not represent a positive McMurray. Distinguish well with the crackling sensation emanating from the patella, often present in patients with femoropatellar OA. |
| Thessaly test at 20 degrees | The participant is in a standing position. Ask the participant to stand on one leg with the knee slightly bent (partial squat, 20 degrees). Take the participant's hands. Take a few side steps to guide the participant towards a rotation of his body. Start to the left, then to the right. Reproduce 3 times without pause on each side. The participant's foot must remain in place on the ground. | **Positive:** if the participant complains of pain / discomfort at the joint space or a feeling of blocking or grasping (snagging). Distinguish well with previous pain which is often present in patients with femoropatellar pathology. |
| Specific tests for femoro-patellar syndrome and / or patellar instability | |  |
| Pain on compression / mobilization of the patella | The participant is in supine position. Push the patella directly against the trochlea. Perform lateral and infero-superior translations (mobilizations). | **Positive:** if the test reproduces the consultation pain. |
| Clarke's test (Patellar grind test) | The participant is in supine position. Wrap the upper aspect of the patella with your hand so that the patella resistance is upwards. You can stabilize the lower limb with your other hand. Ask the participant to contract the quadriceps. | **Positive:** if the participant complains of pain / discomfort representative of Sy. This test creates a lot of false positives in the general population. Run on the healthy knee to make sure it is a true positive (upper pain on the affected side). |
| Patellar apprehension test (30°) | The participant is in supine position. The lower limb is passively placed at 30 degrees of flexion. Exert lateral force on the medial aspect of the patella. | **Positive:** if the participant demonstrates apprehension by resisting lateral force or if the consultation pain / discomfort is reproduced. Compare with the other knee to ensure the validity of the observation. Pain in the external patellar facet does not represent a positive apprehension test. |
| Misalignment (J sign) of the patella during quadricipital contraction | The participant is in supine position. You can stabilize the lower limb with your other hand. Ask the participant to do a quadriceps contraction. Observe the direction of the patella during contraction and / or slightly palpate the upper aspect of the patella to feel the direction of the patella during contraction. You can observe this sign when active knee extension in a seated position, or when walking up and down. | **Positive:** if you observe or palpate a patellar direction with lateral rather than superior contraction. Compare with the other knee to make sure the observation is valid from a clinical utility point of view. |
| FUNCTIONAL ASSESSMENT |  |  |
| Squat analysis | Ask the participant to perform the most complete squat possible. Make sure it is in a safe environment. Note if the participant complains of pain during the execution and / or if he demonstrates a misalignment (ex: valgus, instability) or a limitation of the range of motion, sign of a reduction in motor control. This is a test to assess the functional and overall strength at MI. | |
| Analysis of the ascent and descent of a step | Ask the participant to go up and down a standard stair step (about 25 cm). Make sure it is in a safe environment. Note if the participant complains of pain during the execution and / or if he demonstrates a misalignment (ex: valgus, instability) or a limitation of the range of motion, sign of a reduction in motor control. Compare with the other knee to ensure the validity of the observation. This is a test to assess the functional and overall strength at MI. | |
