# Supplementary material for: Higher psychological distress in patients seeking care for a knee disorder is associated with diagnostic discordance between health care providers: a secondary analysis of a diagnostic concordance study
Source: BMC Musculoskelet Disord. 2021 Jul 30;22:650. doi: 10.1186/s12891-021-04534-9 (PMC8325325; doi:10.1186/s12891-021-04534-9)
Supplement: Supplementary file 2 — Additional file 2. List of analyzed variables. [file 12891_2021_4534_MOESM2_ESM.docx]

**Additional file 2: List of analyzed variables**

**Socio-demographic variables**

Age

Gender

Employment status

Work physical demand

Personal income

**Psychosocial variables**

Kessler 6 total score

Kessler 6 Items (Nervous, Hopeless, Restless, Depressed, Everything is an effort, Worthless)

**Clinical variables**

Duration of symptoms

Onset mechanism (traumatic or progressive)

Timing of symptoms onset if traumatic onset

Timing of apparition of joint swelling if present

Bilateral symptoms

Knee pain location (anterior, posterior, medial, lateral or diffuse)

Use of walking aid

First consultation for this knee disorder

Number of comorbidities (osteoarthritis in other joints, hearth disease, high blood pressure, diabetes)

Height (meters)

Weight (kilograms)

Body Mass Index

MRI results available at the time of consultation

Diagnosis

Number of diagnosis

Knee Injury and Osteoarthritis Outcome Score (KOOS)

KOOS Subscales (Symptoms, Pain, Activities of daily living function, Sports and recreation function, Quality of life)
